# Evaluation of ventilator on lung profile of piglets (*Sus scrofa*) in hypovolemic shock treated with hypervolemic crystalloid resuscitation

**DOI:** 10.14202/vetworld.2019.565-571

**Published:** 2019-04-18

**Authors:** Gunanti Soedjono, Eva Harlina, Antonius H. Pudjiadi, Melpa Susanti Purba, Setyo Jatimahardhiko Widodo

**Affiliations:** 1 Department of Veterinary Clinic Reproduction and Pathology, Division of Veterinary Surgery and Radiology, Faculty of Veterinary Medicine, Bogor Agricultural University, Bogor, Indonesia; 2 Veterinary Paramedic Study Program, Directorate of Diploma Programs, Bogor Agricultural University, Bogor, Indonesia; 3 Department of Veterinary Clinic Reproduction and Pathology, Division of Veterinary Pathology, Faculty of Veterinary Medicine, Bogor Agricultural University, Bogor, Indonesia; 4 Department of Pediatric, Faculty of Medicine, University of Indonesia, Depok, Jawa Barat, Indonesia

**Keywords:** crystalloid, fluid resuscitation, hypovolemia, lung profile, ventilator

## Abstract

**Aim:**

This study was conducted to assess the effect of ventilators on the lung profile of piglets in the hypovolemic shock before and after the excessive resuscitation of the crystalloid fluid.

**Materials and Methods:**

Five male piglets were used in this study as the models of shock, and there are four phases of treatment: Stabilization, shock of bleeding, normovolemic resuscitation, and hypervolemic resuscitation. The application of mechanical ventilation to patients who suspected of having lung injury may worsen the patient’s conditions. The purpose of this study was to set the ventilator with the set of positive end-expiratory pressure (PEEP) of 5 cm H_2_O, thefraction of inspired oxygen (FiO_2_) of 0.5, and the inspiration: expiration (I: E) ratio of 1:2, which was applied from the stabilization phase. The shock induction was performed by removing the blood until the mean arterial pressure decreasing by 20% from the stabilization. The solution of NaCl 0.9% was used for the normovolemic and hypervolemic resuscitation. The parameter of observation consisted of extravascular lung water index (EVLWI) and pulmonary vascular permeability index (PVPI) on pulse contour cardiac output 2 and exhaled tidal volume (VTE), peak inspiratory pressure (PIP), and respiratory rate (RR) on ventilators.

**Results:**

EVLWI does not indicate pulmonary edema. A significant decrease in VTE without any significant alterations in EVLWI, PIP, and RR has indicated the shallow breathing in the shock condition. Therefore, the PVPI parameter cannot be used as a parameter for capillary permeability since its formulation does not reinforce the results of data in the shock condition. The set of the ventilator may prevent the increase of EVLWI, and the uses of ventilators do not worsen the patient’s conditions during the crystalloid resuscitation.

**Conclusion:**

The use of mechanical ventilator as the support does not worsen the hypovolemic condition and is safe to use as long as the lung profile is not indicated to have lung injury.

## Introduction

Shock is a hemodynamic disorder syndrome that often results in death. There are five classifications of shock, namely neurological, cardiogenic, anaphylaxis, septic, and hypovolemic shocks [1]. Hypovolemic shock is a hemodynamic disorder syndrome due to intravascular volume loss of more than 15%, followed by a decrease in perfusion in meeting oxygen and tissue nutrient requirements [2]. The hypovolemic shock can happen due to severe dehydration and severe bleeding [3]. The hypovolemic shock in children at Kenya Hospital in 2013-2016 has a prevalence of 94% with the mortality reaching 34% due to the severe dehydration (diarrhea and vomiting) [4]. According to the World Health Organization (WHO), the mortality rate of pediatric hypovolemic shock in Brazil reaches 800,000 due to diarrhea [5]. The mortality of the hypovolemic shock in children in Indonesia also reaches 6.7% due to severe diarrhea [6].

The severe bleeding from an accident or surgical complications can also cause the hypovolemia, which can develop into the hemorrhagic shock [1]. According to the WHO, the mortality of the hypovolemic shock due to the accidents reaches 5 million people in the World [5]. The first treatment that must be done is the resuscitation of colloid or crystalloid fluid through a central vein [7]. The fluid resuscitation is needed to restore the intravascular volume and to help increase the central venous pressure. Physiologically, if the heart is unable to increase the stroke volume, then the central venous pressure will increase to maintain it. When the hypovolemic shock occurs, the stroke volume is not compensated, so help is needed to increase the central venous pressure [1]. The excessive fluid resuscitation (hypervolemic) can significantly increase the central venous pressure [8]. However, in the septic shock, the excessive fluid administration and the increased central venous pressure correlate with the increased mortality [9]. A fluid resuscitation guide known as the surviving septic campaign in the medical world recommends the resuscitation using the aggressive colloid or crystalloid fluids (*bolus*), as an effort to maximize the cardiac output [10]. However, according to other studies, giving aggressive fluid resuscitation has the potential to damage vascularization and causes edema [11]. The main targeted organ is the one that contains a lot of microvascular like lungs; this increases the possibility to worsen the respiratory system.

The use of mechanical ventilators to support the respiratory system is beneficial to human and animal in critical conditions [12]. However, some researchers report that the use of a ventilator in the lungs can cause acute lung injury (ALI), known as the ventilator-induced lung injury (VILI). This happens if the lungs are indicated to have any damage to the parenchyma and capillaries [13,14]. In Yogyakarta, based on the data from Sardjito hospital, the complications in children with the septic shock reach 88.24% mortality rate. The predictor factors for the cause of death are due to excess of fluid and the use of mechanical ventilators [15]. The gradual fluid therapy can reduce the mortality for ALI [14]. ALI is characterized by an increase in the capillary permeability and pulmonary edema. The effect of the ventilator on the lung profiles of the hypovolemic shock in the young animals before and after the gradual crystalloid fluid resuscitation has not been reported in Indonesia.

This study aimed to assess the effect of ventilators on the lung profile of piglets in the hypovolemic shock before and after the excessive resuscitation of the crystalloid fluid.

## Materials and Methods

### Ethical approval

This research had received the ethical approval from the Animal Ethics Commission of the Faculty of Veterinary Medicine, Bogor Agricultural University with the SKHE No. 055/KEH/SKE/III/2017.

### Materials and tools

The anesthetic preparations used were 10% Xylazine (Ilium Xylazil^®^, Ilium), 10% Ketamine (Ket-A-100^®^, Agrovet), and local anesthetic Lidocaine of 2% (Lidocaine^®^, Phapros). Other ingredients used were Povidone-Iodine (Iodine^®^, OneMed), Alcohol (Alkohol of 70%, AMS), nonsteroidal anti-inflammatory drug flunixin preparation (Flunixin^®^, Norbrook), Oxytetracycline antibiotics (Vet-Oxytetracycline^®^), Gentamicin (Gentalex^®^), anthelmintic Oxfendazole (Vermo-O^®^, Sanbe), and 0.9% NaCl (Ecosol NaCl^®^, B. Braun) as the crystalloid liquid, Sodium Heparin 5000 IU/ml (INVICLOT^®^) as the anticoagulant preparation, and 0.9% cold NaCl (8°C) as thermodilution injectate.

The equipment used in this research were the scales, a set of minor surgical tools, the surgical blade (GEA^®^-Medical), the chromic 3/0 (GEA^®^-Medical) catgut, the silk thread of 3/0 (DR. SELLA^®^), 5-mm diameter endotracheal tube (ETT), the laryngoscope, the micropore, the infuse set, the syringe of 1, 3, 5, 10, and 50 ml, three-way stop cock, intravenous (IV) 22G catheter (Certofix^®^, B Braun), the oxygen cylinders, the oximetry pulse, the mechanical ventilator (Ventilator^®^, Hamilton Galileo Gold), a set of pulse contour cardiac output (PiCCO) Plus v4.12 (PiCCO_2_^®^, Pulsion Medical Systems AG), the catheter of PiCCO (Pulsiocath^®^, Pulsion Medical Systems AG), and the patient monitoring setup that is used to determine the physiological state of animal models in the real time.

### Animal acclimatization

Five healthy male piglets (*Sus scrofa*) aged 6-10 weeks, weighing 10-20 kg, were treated according to the standard management on the Faculty of Veterinary Medicine, Bogor Agricultural University. The animal acclimatization was done in the Laboratory Animal Management Unit for 15 days. The animals were given morning and evening feed and *ad libitum* drinking. During the acclimatization, the animals were given the antibiotics of the oxytetracycline at a dose of 10 mg/kg body weight (BW) parenterally the intramuscularly (IM) route and the worm drugs of the oxfendazole in *bolus* mixed with the feed. The animal cages were cleaned in the morning and evening. The healthy animals were weighed before being made into the animal models.

### Research phases

This research consisted of two phases, namely the preparation and treatment phase. The preparation phase consisted of the anesthesia, installation of the mechanical ventilator, and installation of PiCCO^®^. The treatment phase consisted of stabilization to maintain the normal condition, the bleeding phase for the insistence of the hypovolemic shock, the normovolemic phase, which is the injection of crystalloid as much as the volume of blood fluid lost, and the hypervolemic phase of the crystalloid injection for 40 ml/kg of BW.

#### Preparation phase

The preparation phase began with the anesthesia of the animals in the cage using 20 mg/kg of 10% ketamine and 2 mg/kg of 10% xylazine IM in the femoral area. After the animal was anesthetized, the animal was weighed to help calculate the next anesthetic dose, and the BW value was used in measuring the variable value.

The animal was cleaned by shaving the hair and giving antiseptics and povidone-iodine to the cervical and femoral orientation. The installation ofIV catheter and three-way stop cock was performed on the auricular vein, which was used to access the infusion and injection of the anesthetic preparation. Then, the animal was moved into the operating room, followed by the installation of the tomfool restrain mode on the operating table. The installation of 0.9% NaCl infusion was connected to the IV catheter that was previously installed. The pulse oximeter was mounted on the tail of the animal model.

Procedure for installing the mechanical ventilator

Before the installation of the mechanical ventilator, the animals were given lidocaine topically in the epiglottis. The installation of the ETT was assisted using a laryngoscope, which was then connected to the mechanical ventilator. The ventilated animals were controlled in the ventilation set of 10 ml/kg tidal volume, PEEP of 5 cm H_2_O, the FiO_2_ of 0.5, and the ratio of the I: E of 1:2. This arrangement was the gold standard for healthy lungs. Other variables such as the exhaled tidal volume (VTE), the respiratory rate (RR), and the peak-inspiratory pressure (PIP) were used as the readers’ values for changes in the lung ventilation during the treatment.

Installation procedure of PiCCO^®^

The installation of PiCCO^®^ began with the small operation looking for the vein and arterial accesses for the catheter of the device sensor of PiCCO^®^. This sensor was installed by connecting the catheter into the jugular vein and thermistor into the femoral artery. The vascular access search was carried out by removing the muscles of the right medial cervical region for the jugular vein and the left femoral medial for the femoral artery. After the access was obtained, the catheter of PiCCO_2_^®^ was installed, and then, the antibiotics and temporary sewing were performed. The catheter was also used as an access to the cold thermodilution injectate (<8°C) in *bolus* of 10 ml. The anesthesia period was extended with the addition of the ketamine-xylazine (4:1) 1 ml manually through the auricular vein.

#### Treatment phase

The treatment phase consisted of four procedures, namely the stabilization, the bleeding, the normovolemic resuscitation, and the hypervolemic resuscitation.

Stabilization

After all the tools were installed, the animal was maintained in a stable condition. After giving the cold *bolus* (8°C) on a *bolus* basis, the CO curve will form on the monitor of PiCCO. The *bolus* insertion was performed twice to obtain the initial hemodynamic data or the baseline.

Procedure for seeking hypovolemic shock

The bleeding phase was intended to make a hypovolemic shock. The indicator of shock in this study was marked by the decrease in the mean arterial pressure (MAP) of 20% from the stable condition. The blood was removed using the 50-ml syringe and released through the jugular vein into a container. The bleeding phase was done until the MAP reaches the shock indicator. The shock condition was maintained for 15 min, and the hypovolemic shock data were collected.

Normovolemic resuscitation procedure

The normovolemic resuscitation was done by injecting crystalloid fluid as soon as possible using the 50-ml syringe through the jugular vein as much as the volume of blood released, and then, the normovolemic data were taken.

Hypervolemic resuscitation procedure

The hypervolemic resuscitation phase was carried out by the injection of crystalloid fluid using the 50-ml syringe through the jugular vein with the volume of 40 ml/kg BW, and then, the hypervolemic data were collected. The data retrieval was done 3 times at each phase with a span of 3 min to take the average value. The time interval needed for each treatment was 30 min. The anticipation of the vascular hemostatic event was carried out by entering 0.9% NaCl liquid mixed with the sodium heparin of 5000 IU through the jugular vein access.

### Observation variable and data analysis

The variables observed in this study were the extravascular lung water index (EVLWI) and the pulmonary vascular permeability index (PVPI) obtained from the PiCCO^®^ and the PIP, RR, and VTE obtained from the mechanical ventilator as the lung monitoring. The focus of the research lies in the changes that occur during the treatment and was reviewed at each phase of the treatment. The data processing phase was carried out using SPSS Version 16 (IBM, USA) with the paired sample t-test analysis method. The analysis results were expressed in the form of the mean±standard of deviation.

## Results

### EVLWI

The parameter of the EVLWI is a parameter which describes the state of the fluid in the interstitial space and pulmonary alveoli. EVLWI is often used to represent the severity of the pulmonary edema. The normal EVLWI value in the adult pigs is 16.3±5.2 ml/kg [16], whereas, in the piglets, it is 14.77±4.31 ml/kg [17]. The data of EVLWI (Table-1) show the non-significant decrease and increase between the treatment phases.

**Table-1:** Mean of EVLWI (ml/kg) before and after resuscitation.

Data retrieval time	Mean of EVLW
Beginning	15.2±6.8^a^
Hypovolemia	13.3±5.4^a^
Normovolemic resuscitation	13.5±5.2^a^
Hypervolemic resuscitation	15.4±5.4^a^
Hypervolemic after 30 min	15.1±5.7^a^

Different superscript letters show the significant differences (p<0.05) between the data retrieval time, EVLW=Extravascular lung water index

The parameters of EVLWI, PVPI, and VTE describe the profile of the level of damage from the lungs. The data of EVLWI show that there are no significant differences (p>0.05) at all treatment times. This explains that the condition of the lungs does not experience an increase in the lung fluid which indicates the pulmonary edema. The value of EVLWI is still in the normal range according to the literature, both in the normal value of the adult pigs of 16.3±5.2 ml/kg [16] and the piglets of 14.77±4.31 ml/kg [17]. The value that indicates the occurrence of the edema in the pigs of the ALI is 19.2±2.8 ml/kg [18], whereas, according to the results of other studies, the value that indicates the occurrence of the edema in the pigs of the septic shock model is 22.00±2.16 ml/kg [17].

### PVPI

The parameter of PVPI represents the ratio between the fluid in the lung space and the vascular blood volume. This ratio describes the vascular permeability in the pulmonary capillaries. The calculation of this ratio is obtained from the value of EVLWI divided by the pulmonary blood volume (PBV). The normal PVPI value in the piglets is 3.35±0.31 [17]. The data of PVPI (Table-2) show a significant decrease (p<0.05) at the normovolemic time. The data of PVPI show an increase that is not significantly different (p>0.05) at the time of the hypovolemic shock and a significant decrease (p<0.05) during the normovolemic resuscitation. However, PVPI data of the hypervolemic resuscitation time after 30 min show that the increase of PVPI (p>0.05) accompanied by the increase of EVLWI (p>0.05) and the value in the normal range. This indicates that the application of the hypervolemic resuscitation does not cause vascular and endothelial damage. However, the crystalloid fluids have the small molecules, so there is an increase in the hydrostatic pressure caused by the resuscitation procedure, pushing the fluids easily through the capillary membrane to the interstitial [19]. The increase in the pulmonary extravascular fluid is thought to be due to the hypervolemic resuscitation procedures that trigger increased intravascular hydrostatic pressure, small molecular size of crystalloid fluid, and lack of positive ventilator pressure.

**Table-2:** Mean of PVPI before and after resuscitation.

Data retrieval time	Mean of PVPI
Beginning	3.25±0.5^a,b^
Hypovolemia	3.52±0.1^a^
Normovolemic resuscitation	3.05±0.2^b^
Hypervolemic resuscitation	3.10±0.5^a,b^
Hypervolemic after 30 min	3.25±0.4^a,b^

Different superscript letters show the significant differences (p<0.05) between the data retrieval time, PVPI=Pulmonary vascular permeability index

### VTE

The tidal volume is the volume of air entering and leaving the lungs. The parameter of VTE describes the volume of air exhaled by the lungs. The value of VTE is quite important because it represents the actual volume of air that expands in the lungs. The normal range of tidal volume in the pigs is 5.9-14.5 ml/kg [20]. The data of VTE (Table-3) show a significant difference in the initial observation of the hypovolemic condition.

**Table-3:** Mean of the VTE (ml/kg) before and after resuscitation.

Data retrieval time	Mean of VTE
Beginning	12.5±1.2^a^
Hypovolemia	11.3±1.2^b^
Normovolemic resuscitation	11.5±1.7^a,b^
Hypervolemic resuscitation	11.5±1.5^a,b^
After 30 min	11.0±2.1^a,b^

Different superscript letters show the significant differences (p<0.05) between the data retrieval time, VTE=Exhaled tidal volume

The data of VTE have a correlation with the increase in the values of RR which are not significantly different when the hypovolemic shock occurred. The VTE value has decreased which is significantly different (p<0.05), indicating that there is an increase in the number of the respiration rate when it is hypovolemic. This value represents the short and shallow breaths. This allegation is reinforced by other researchers who state that when the body experiences the blood loss to reach more than 15%, then the body will experience the increase in the heart rate and the increase in the respiration rate [21]. However, the absence of the significance of the data of RR refers to thenonoptimal shock efforts.

The value of EVLWI after the hypervolemic resuscitation is in the normal range and indicates that the alveoli space has no edema. This is evidenced by no significant difference in the hypervolemic VTE data (p>0.05). VTE represents the volume of the air contained in the alveoli.

### RR

The parameter of the RR is widely used as the important data from the actual number of breaths needed by the patient. The respiration rate can change depending on the patient’s needs. The normal value of RR of young male pigs is 37±12 times/min [22]. The data of RR (Table-4) show that there is an insignificant increase at the baseline to the hypovolemic conditions. This shows a correlation between RRs with a decrease in VTE that is significantly different (p>0.05), so it can be concluded that the animals experience rapid and shallow breathing rates.

**Table-4:** Mean of RR (time/min) before and after resuscitation.

Data retrieval time	Mean of RR
Beginning	32.8±7.6^a^
Hypovolemia	33.7±7.0^a^
Normovolemic resuscitation	33.7±5.0^a^
Hypervolemic resuscitation	33.5±5.1^a^
Hypervolemic after 30 min	33.8±6.1^a^

Different superscript letters show the significant differences (p<0.05) between the data retrieval time, RR=Respiratory rate

### PIP

PIP is a parameter used to observe the highest level of the pressure that has been achieved every sniff. The normal PIP value in the piglets is 18.6±1.5 cm H_2_O [18]. The PIP data (Table-5) show that the value decreasing non-significantly during the shock observation.

**Table 5:** Mean of the PIP (cm H_2_O) before and after resuscitation.

Data retrieval time	Mean of PIP
Beginning	22.0±7.3^a^
Hypovolemia	18.4±1.7^a^
Normovolemic resuscitation	19.4±2.9^a^
Hypervolemic resuscitation	18.0±1.4^a^
Hypervolemic after 30 min	19.0±1.2^a^

Different superscript letters show the significant differences (p<0.05) between the data retrieval time, PIP=Peak inspiratory pressure

## Discussion

### EVLWI

The non-significant decrease in the value of EVLWI (p>0.05) is caused by the pulmonary extravascular fluid undergoing the redistribution intravasation. This can be caused by three possibilities. First, there is a difference in the hydrostatic pressure decreasing compared to extravascular. Increased value of the intravascular hydrostatic and decreased value the extravascular hydrostatic are determinants of the pulmonary edema [23]. Second, the role of the ventilator set helps to redistribute the extravascular fluid. The role of the ventilator as a distributor of the positive pressure in preventing the alveolar collapse also helps to redistribute the extravascular fluid and to reduce the chance of the edema [24]. The extravascular fluid redistribution results from the positive pressure that the ventilator delivers to the alveolar and interstitial spaces, thereby suppressing and preventing the extravascular intravascular fluid. Third, it can also caused by the increasing reabsorption by the lymphatic system. The compensation of the body homeostasis will increase the reabsorption of the extravascular fluid by the lymphatic system when the intravascular fluid volume decreases and the fluid distribution returns to the vein [25].

### PVPI

The increase of PVPI is caused by the bleeding process. Physiologically, the presence of immune activity, cytokines, and several other mediators due to the bleeding allows the changes in the capillary permeability [26]. There is another research comparing the effects of crystalloids on Beagle hemorrhagic shock that also shows an increase in PVPI during shock and decreased PVPI during the normovolemic resuscitation [27]. However, there is no literature that explains physiologically why the value of PVPI decreases after the post-shock (normovolemic) resuscitation. Mathematically, the value of PVPI is calculated as the ratio between EVLWI and PBV so that this literature can answer other guesses why this value rises during shock and falls significantly when the crystalloid fluid normovolemic resuscitation is performed [28].

The increase of PVPI value without an increase of EVLWI during the hypovolemic shock does not indicate that the lungs have the ALI. PVPI and EVLWI values that indicate ALI in ALI model pigs are 5.9±1.3 and 19.2±2.8 ml/kg, respectively [18].

### VTE

When a decrease in tidal volume is blown, the lungs have decreased capacity to hold the air. This assumption can refer to a state of edema, alveoli leakage, and atelectasis. The alveoli leakage begins with the barotrauma, namely the parenchymal damage due to the overdistention by the high-pressure ventilators. The barotrauma often occurs and is associated with the VILI [29,13]. Atelectasis results from the obstruction and loss of positive thoracic cavity pressure, causing the collapse of the alveoli [29]. The parameter of the PIP can be used as the barotrauma parameters, and the use of the set ventilator of the PEEP can also be associated with the significant changes in VTE (p<0.05) in the conditions of the hypovolemic shock.

### PIP

The value of PIP indicates the barotrauma if there is a significant increase of PIP [30]. The high PIP value at the beginning of the data is a result of adjusting the ventilator set used with the readiness of patients to receive artificial ventilation. This is reinforced by other researchers that the high pressure channeled by the ventilator is caused by the initial adjustment of the ventilator set to the patient’s spontaneous breathing, after which it reaches the synergistic breathing with the ventilator [18,29]. The PIP correlates with the use of the PEEP. If the ventilator set uses the high PEEP >7, it can cause barotrauma and trigger the decrease in the pulmonary compliance which is an expansion of lung elasticity, the tidal volume representing the expanding air capacity, the outflow of CO_2_, and the cardiac output, while the use of low PEEP <3 can trigger the atelectasis [29]. In this study, the ideal set of PEEP prevents the alveolar collapse and does not cause the overdistention. The patients are more comfortable breathing spontaneously when receiving the ventilation with the ideal pressure and volume [31].

The use of the ventilator does not worsen the condition both during the hypovolemic shock and 30 min after the hypervolemic resuscitation. This is indicated by the parameter data of PIP and RR which are not significant. The patients with artificial hypovolemic shock can be corrected by the hypervolemic crystalloid resuscitation. This is indicated by the parameter data of EVLWI. The parameter of VTE shows the marked decrease as a result of the pursuit of the hypovolemic shock. Without the marked increase in the RR, the pursuit of the hypovolemic shock is mild. The parameter of PVPI cannot be used as the reference for measuring the vascular permeability when used in the conditions that affect the calculation of formulation such as the hemorrhagic shock condition and the resuscitation of the hemorrhagic post-shock.

## Conclusion

The use of mechanical ventilator as support does not worsen the hypovolemic conditions and is safe to use as long as the lung profile is not indicated to have lung injury.

## Authors’ contributions

GS, EH, and AHP designed and coordinated the study. GS supervised the present study. GS, EH, AHP, MSP, and SJW performed the experiment. GS, EH, and SJW analyzed the data and wrote the manuscript. The final manuscript has been read and developed in consultation with all authors. All authors read and approved the final manuscript.
